# Going Mobile: How Mobile Personal Health Records Can Improve Health Care During Emergencies

**DOI:** 10.2196/mhealth.3017

**Published:** 2014-03-05

**Authors:** Nidhi Bouri, Sanjana Ravi

**Affiliations:** ^1^UPMC Center for Health SecurityBaltimore, MDUnited States

**Keywords:** electronic health record, personal health record, public health emergency, mobile health

## Abstract

Personal health records (PHRs), in contrast to electronic health records (EHRs) or electronic medical records (EMRs), are health records in which data are accessible to patients and not just providers. In recent years, many systems have enabled PHRs to be available in a mobile format. Mobile PHRs (mPHRs) allow patients to access health information via the Internet or telecommunication devices, such as mobile phones, personal digital assistants, and tablet computers. mPHRs have the potential to help patients and providers identify medical conditions and prescriptions from numerous locations, which may minimize medical errors and identify improvements to health behaviors during emergencies, when patients present to a new provider, or EHRs are not accessible. Despite their benefits, numerous challenges inhibit the adoption and further development of mPHRs, including integration into overall health technology infrastructure and legal and security concerns. This paper identifies the benefits of mPHRs during emergencies and the remaining challenges impeding full adoption and use, and provides recommendations to federal agencies to enhance support and use of mPHRs.

## Introduction

The use of electronic records has been widely recognized as an efficient way to improve the provision of health care and enable health care providers to access and share patient information. Health care providers may document a patient’s medical history in a number of ways. Electronic medical records (EMRs), for instance, are digital copies of patient charts commonly used in physicians’ offices to record patient data [1]. Electronic health records (EHRs) are more comprehensive in scope, including information from all the clinicians involved in a patient’s treatment, such as immunizations, family medical histories, and previous providers [1]. Primary care physicians may even share EHRs with other health care professionals and institutions, including specialists, laboratories, and nursing homes [1]. The information documented in EHRs is also found in personal health records (PHRs). However, unlike EHRs, which are only accessibly to clinicians, patients can use PHRs to manage and update their own medical information [1]. PHRs can therefore empower individuals to improve their health status and improve clinical outcomes, as they are able to better monitor health conditions and more effectively communicate with health care providers. Patients typically use PHRs in one of three formats: a provider-maintained digital summary of clinical information accessible to patients; a patient-owned software program that allows users to view and update their own health information; or portable, interoperable digital files with which patients can manage and transfer information [2]. PHRs in mobile format (mPHRs) fall into the third category and allow patients to access health information via the Internet or telecommunication devices, such as cellular phones (specifically, smartphones, or cellular phones that includes an operating system capable of running general-purpose applications and performing many of the functions of a computer), personal digital assistants, and tablet computers. The increasing use of mPHRs among patients reflects a broader trend in health care digitization: the growing popularity and utility of mobile medical applications [3]. Such applications operate on the aforementioned mobile devices, which have rapidly evolved into ubiquitous tools for sharing information and communicating with others. Mobile devices also possess the potential to withstand certain types of infrastructural failures during disasters. As such, they may be uniquely qualified to play important roles in responding to public health emergencies (PHEs).

To date, studies indicate that PHRs and their mobile counterparts have served patients well in their interactions with health care professionals, but these studies focus primarily on the applications of PHRs during non-emergencies [4-8]. Similarly, consumers and health care practitioners thus far have used PHRs largely in nonemergency settings. However, the incidence of PHEs—specifically, natural disasters—is increasing worldwide [9]. Correspondingly, the number of individuals who are injured or displaced by such events is also likely to rise, thereby generating major logistical challenges for health care delivery in post-disaster settings. In such situations, reliable sources of clinical information are invaluable to patients who cannot communicate or receive treatment from caregivers who are unfamiliar with their medical histories. Given the challenges associated with communicating during disasters, we suggest that integrating PHRs and mPHRs into emergency response plans could help ensure quality health care delivery if or when existing methods of information sharing (eg, paper- and/or computer-based records) fail. However, several critical challenges must first be addressed.

To support the analysis in this article, we conducted a literature review using the research databases PubMed and SCOPUS. We then identified scientific studies and peer-reviewed literature that address PHRs and their use in emergency and nonemergency settings, as well as legal and regulatory concerns relating to their use. We also reviewed literature from federal agencies, particularly the Department of Health and Human Services (HHS), regarding health information technology (HIT) and EHRs, and the potential integration of PHRs into these processes.

In this paper, we first provide an overview of state of the art research regarding PHRs and their various applications. Based on state of the art research and secondary sources (eg, reports from federal and private entities), we next consider the entities involved in mobile health innovation in the United States. Third, we explore the applications of mPHRs, along with their associated strengths and shortcomings, as well as the legal and regulatory frameworks for HIT in the United States. Finally, we conclude with policy recommendations to clarify the roles of PHR technologies in emergency response and improve post-emergency health care delivery.

## PHR Applications

### Overview

In recent years, scientific studies have documented the role of PHRs in improving self-care practices among patients and transforming health care delivery [10]. Studies have looked at the various formats of PHRs, including Web-based and mobile-based applications, as well as the use of PHRs among specific populations, such as patients with chronic health issues and children. Moreover, the growth of self-management tools for remote monitoring, particularly those available in Web and mobile application formats, contributes to the increased use of PHRs and mPHRs among consumers. Similarly, providers’ perceptions that PHRs are useful self-monitoring and information-sharing tools can permit PHRs to play a key role in ensuring continuity of care [11-14].

Several studies indicate that PHR tools can improve clinical outcomes. For example, PHR use has been associated with improved self-monitoring and positive clinical outcomes for hypertension [15], adherence to immunizations and other practices supporting child wellness [16], and management of medications such as medication review and hypertension monitoring tools [17]. However, studies also indicate that the benefits of PHRs in improving clinical outcomes can be correlated with age, because younger patients are more likely to use PHRs frequently. Ethnic and racial minorities have also been reported to adopt PHRs less frequently than whites do, and patients from lower income groups are less likely to use PHRs as compared to those with higher incomes [18]. Previous studies have also addressed barriers and concerns to the use of PHRs and, more generally, personal health information. Such concerns include access to safeguarding patient privacy [19] and emergency access to patient-controlled online health data [20].

While the various uses of PHRs and factors influencing their development have been well documented in the scientific literature, there is limited literature assessing the unique role and challenges of mPHRs or the role of PHRs and mPHRs during emergencies. Furthermore, there are no documented uses of PHR technologies during disasters. Still, the positive clinical outcomes achieved through PHR use may be replicated in emergencies with the appropriate infrastructures and policies in place. Below, we explore the current state of PHR development, use, and regulation in the United States, highlighting areas requiring further attention.

### Key Players

#### Overview

Several entities are involved in the collection, storage, and dissemination of personal health information. Of these, consumers, technology developers, health insurance companies, and federal agencies play particularly important roles. As HIT comes to play a greater role in emergency response and recovery, it is imperative that policies address the needs of stakeholders from each of these groups, particularly in matters of patient care. This is especially important, given the need for more information on the ways emerging technologies might address the unmet needs of special populations (eg, children, the homeless, disabled individuals, non-English speakers, etc). Below, we identify the various impacts that these groups have had on PHR and mPHR development and use, and remaining concerns to be addressed.

#### Consumers

A 2012 study from the Pew Research Center concluded that 85% of US adults own a cell phone, 53% of whom own smartphones [21]. Approximately one-third of cell phone owners (31%) have used their phones to look for health information, which is a 17% increase from a national-level comparable survey conducted in 2010 [21].The study also indicated that 52% of smartphone owners gather health information on their phones, compared to only 6% of non-smartphone owners [21]. Cell phone owners who identify themselves as Latino, African American, between the ages of 18 and 49 years, or hold a college degree are also more likely to gather health information using their cell phone [21]. Furthermore, approximately one-fifth of smartphone owners (19%) have at least one health application on their phone. Marketing forecasts indicate that by 2017, half of the world’s 3.4 billion smartphone or tablet users will use mobile health apps [22]. As the use of mobile health applications continue to grow, particularly among specific demographic groups, many entities should consider how mPHRs could be developed to support emergency and post-disaster care, in addition to routine health management.

Consumers, health insurance companies, and mobile application developers have all generated support for PHRs, albeit inconsistently. A 2011 survey of 1200 respondents conducted by IDC Health Insights revealed that 7% (84) of consumers had created online PHRs, but fewer than half continued using them [23]. However, other providers report significantly higher rates of customer retention and PHR usage. In 2011, for example, Kaiser Permanente’s PHR service, My Health Manager, saw 74 million logins, 10 million prescription refills, and 29.7 million lab results viewed, indicating frequent patient-provider interactions [24].

Similarly, certain populations—notably, disabled individuals—have expressed interest in portable PHR solutions such as smart cards or implantable microchips for medical emergencies [5]. One survey of 302 individuals (categorized as “well,” “unwell,” and “disabled”) reported that several nondisabled respondents indicated they had thought about how their information would be accessed in an emergency. However, only disabled respondents described incidents where access to personal medical data rose to the level of a life-or-death issue, which is consistent with disabled individuals’ overall higher level of emergency department (ED) utilization [5]. Interestingly, health-related computer use (ie, searching for medical information, communicating with providers, and filling prescriptions) among disabled populations is 19% higher than that among nondisabled individuals, signifying a potential niche for the expansion of PHR services [5]. Homeless individuals represent another population that could benefit greatly from increased PHR use. Recent research indicates that homeless patients make considerable use of ED services and are significantly more likely to solicit information on chronic health problems than patients with stable housing [25]. A study of more than 5700 ED visitors revealed that 70.7% (176/249) of homeless patients own cell phones (compared to 85.9% of patients with stable housing) [25]. Furthermore, there was no significant difference in the frequency of new media use between these two groups [25].

In light of the failure of now-defunct PHR services such as Google Health and Revolution Health, it follows that successful consumer technologies are those that “inform or entertain users, or enable social communication” [23]. Other groups—notably, Microsoft, Kaiser Permanente, and WebMD—have enjoyed greater popularity among consumers by partnering with insurance companies and other health care providers, a strategy that is successful because it integrates new technologies into existing platforms and business models [23]. Similarly, in a 2008 study conducted by User Centric in which subjects were asked to build their own PHRs, the majority of participants were interested in maintaining them after the study [26]. The move toward digitization, combined with growing numbers of smartphone and social media users, empowers consumers to more proactively monitor their health. As technologies that facilitate this shift, mPHRs could evolve into important elements of today’s HIT infrastructure.

#### PHR Developers

Despite their status as emerging technologies, PHRs—both mobile and Web-based—already come in many varieties and address numerous medical conditions. Because they enable patients to track their health conditions outside of a medical facility, PHRs and mPHRs boast a variety of applications in chronic and noncommunicable disease management, particularly for ailments like diabetes mellitus, heart disease, cancer, and mental illness [8]. For example, BodyKom, a mobile application for cell phones, enables nonhospitalized cardiac patients to track and share their vital signs with physicians via their cell phones [27]. Dexcom has also developed a biosensor that monitors blood sugar levels, transmits data to patients’ mobile devices, and automatically updates their mPHRs as well as their providers’ EHRs [8]. Several mental health applications (eg, Mobile Therapy and CBT MobilWork) function as ad-hoc therapy tools, enabling patients to record symptoms between clinician visits and providing mental health professionals with a plethora of information on their patients’ health statuses outside of medical settings [7]. Given the increasing use of EMRs and EHRs, electronic PHRs and mPHRs in particular represent an important development in HIT.

#### Insurance Companies

Given that insurance claims are an important source of medical information, health insurance providers represent another major stakeholder in the expansion of PHR services. Medical practitioners have also shown support for PHRs, particularly in situations involving patient transfer between caregivers [28]. A 2009 national survey reveals that 42% of doctors—especially those practicing in rural areas—are willing to start using electronic PHRs [29]. Companies like Blue Cross and Blue Shield, Aetna, and United Healthcare all offer PHR tools to their clientele; of these, Aetna also offers a mobile option for accessing personal health information.

During the aftermath of Hurricanes Katrina and Rita, medical professionals faced a major health care delivery challenge: obtaining accurate medical histories for the 7500-plus individuals who were ill, injured, or seeking consultation [30]. In response, Blue Cross and Blue Shield, which reported that over 300,000 customers and providers lost their medical records, began offering claims-based health histories to evacuees receiving medical treatment from new caregivers [31]. Hurricane Katrina also prompted the Markle Foundation to implement a different solution to the problem of health care delivery: an online portal called KatrinaHealth, where patients, pharmacists, and doctors could easily access prescription and dosage records in the wake of the storm [32]. System users reported that ready access to this information helped evacuees renew their medications and assisted health care professionals in coordinating care and avoiding prescription errors [33]. The Markle Foundation further reported that electronic PHRs could one day eliminate the logistical barriers associated with maintaining decentralized sets of patient records, but “are not yet a viable solution in disasters…because too few people have them and they exist on multiple platforms that may not be compatible with one another” [32]. Furthermore, because authorities have granted little attention to the management of personal medical information in emergencies, concerns over privacy and security continue to dissuade American consumers from using PHRs [32].

#### Federal Agencies

Despite support from consumers, insurance companies, and health care professionals, the federal government has offered relatively little guidance and made limited progress in integrating PHRs into the nation’s existing HIT infrastructure. However, a select few federal agencies, such as the Centers for Medicare & Medicaid Services, have recognized the potential of PHRs and support their use to improve patient-provider communication [34]. HHS has also released a privacy notice for PHRs supporting “individuals’ use of PHRs as a mechanism to facilitate access to, and control over, their health information” [35]. The Centers for Disease Control & Prevention (CDC) have supported HHS’ work on PHRs, calling for further research on confidentiality, security, interoperability, regulation, and evaluation [6]. The US Food and Drug Administration (FDA), meanwhile, which maintains regulatory responsibility for medical devices, has formulated policies pertaining to certain mobile health applications. These policies, however, do not extend explicitly to EHR or PHR applications [36]. In September 2013, the FDA released a guidance document stating that it would exercise “enforcement discretion for mobile apps that enable patients or providers to interact with Personal Health Record or Electronic Health Record systems” [37]. The document goes on to specify that in addressing these applications, the FDA will not enforce the mandates established by the Food, Drug, and Cosmetic Act [37].

Considering the roles that PHRs (including mPHRs) might play during PHEs, increased involvement from consumers, insurance companies, clinicians, and federal entities is necessary. Engaging other stakeholders—emergency responders, medical volunteers, and public health laboratories, for instance—is also critical to the successful integration of PHRs into emergency response efforts.

## Benefits

### Examples

Just as the shift from paper-based records to EHRs provides numerous benefits to providers and patients, mPHRs can also capitalize on the benefits of digital records. In fact, mPHRs arguably provide more benefits to patients than EHRs, as increasing and widespread Internet access and mobile device use allow patients to access their records from anywhere with an Internet connection. PHEs typically present emergency responders with immense logistical challenges with respect to exchanging information in a timely and accurate manner, particularly in health care settings [38]. In such situations, when the displacement of an affected community can impede access to routine providers, alternate modes of communication are necessary to ensure continuity of care. PHRs—and mPHRs in particular—may present a viable solution to this recurring problem. Although mobile-based information exchange systems themselves are not immune to failure, they nevertheless present patients and clinicians with a feasible backup strategy for sharing critical medical information. In fact, many health care practitioners who treated patients following Hurricane Katrina supported the use of standardized, interoperable, electronic PHRs as a means of enhancing emergency patient care [39].

### Proven Benefits

#### mPHRs Allow Health Care Providers to Better Share Information With Patients

mPHRs give providers a mechanism to share information with patients, including clinical summaries, diagnoses, educational resources, and appointment reminders. They also enable patients to refill prescriptions, access lab results, track immunizations, and schedule appointments. Some of these features (eg, prescription refills) exist in applications developed by major retail outlets such as Walgreens and CVS, but do not comprise a holistic health record. However, other mobile services (eg, Group Health, Castlight Mobile, MyChart, myCigna, Coventry Mobile, MHBPSM Mobile, Evita Personal Health Record, and Capzule PHR) do serve as holistic records of health information. Still other applications—such as Health4Me and IBX—even enable patients to view their insurance claims, track health spending, and search for local health care professionals. As indicated in one study, the functions of PHRs have the potential to create a more comprehensive and balanced view of the patient, because patients control and manage the information in the record. Health care providers can therefore view records, as documented by patients, directly [11].

PHRs thus allow patients to reference pertinent medical information at any time and share such information with other providers, even during ED visits and other unscheduled visits. This feature of PHRs also facilitates continuity of care if a patient receives treatment from a new provider. The clinician in this case would have access to a thorough record of the patient’s existing medical conditions, including previous medical tests, procedures, prescriptions, and conditions. Such information, in turn, would prevent duplication of tests and treatments and minimize risk of administering medications that can complicate conditions or allergies.

There are several mPHRs that serve many of the aforementioned functions; some are even specially customized for emergencies. Microsoft HealthVault, for example, enables users to create medical records specifically for unexpected hospital visits or to inform first responders. Another application, Gazelle, allows smartphone users to receive and share lab results. Similarly, a Web-based PHR service called Synchart stores patients’ health information and can grant clinicians emergency access to said information during a crisis.

### Potential Benefits

#### mPHRs Provide Patient Medical Histories at the Point of Care

mPHRs, which may improve provision of care during PHEs, give health care providers instant access to a patient’s medical history and recent medical events that can be beneficial in both emergency and nonemergency situations. However, PHEs, which may encompass events as diverse as infectious disease outbreaks, natural disasters, or manmade catastrophes, present logistical barriers that impede health care delivery and timely access to critical medical information. During PHEs, when many patients are displaced or health care facilities lose abilities to access EHRs, mPHRs may be one of the only ways of providing accurate, recent medical information. Patients will likely present to makeshift medical facilities or those with limited operational capabilities, receive care from an unknown provider, or be unable to communicate their medical histories.

When patients are unable to seek care from their primary care physician or a facility with access to their medical history, mPHRs can help providers determine essential information, such as medical conditions and drug allergies, needed to inform treatment options and better coordinate and direct care. Such information could be useful when health systems are strained and facilities may lack adequate numbers of staff. mPHRs can inform providers of important health information that can ultimately reduce medical error and improve triage. mPHRs may also particularly benefit specific populations during PHEs, including nonresponsive patients who cannot communicate with providers, those who seek care at an alternative care site, vulnerable and special populations, and children and young adults. For vulnerable and special populations, such as non-English-speaking persons, those belonging to ethnic minority groups, mentally unstable patients, and those who are deaf or blind, mPHRs may be the only communication method between patient and provider. Smartphone ownership and broadband Internet access among many of these populations is increasing. Nationally, between 2000 and 2011, rates of Internet access increased among Hispanic, Black, and Asian households by 34.7%, 33.3%, and 26.5%, respectively [40]. Similarly, as of 2013, 51.6% of Asians report owning a smartphone, along with 48% of African Americans and 45.4% of Hispanics [40]. Among Latino Internet users, 28% are predominantly Spanish speakers, suggesting that a Web-based PHR platform may be an effective way of reaching this target minority group [41].

Pediatric populations can also present unique challenges to providing health care in emergency settings, particularly when children either do not have or are separated from parents and guardians. In California, for example, there are more than 56,000 children in foster care, many of whom face barriers to health care access despite greater needs (50% of foster care children suffer from at least 1 chronic condition, while 25% suffer from 3 or more) [42]. These patterns also hold nationally, where 90% of children entering foster care have physical health problems and another 55% suffer from 2 or more chronic conditions; these individuals also make more frequent use of costly medical services such as group care, inpatient psychiatric treatment, and ED services [42]. mPHRs will likely benefit clinicians serving this particular population, given the increasing use of smartphones among teenagers and young children. In March 2013, a national survey estimated that 37% of American teens (ages 12-17 years) own a smartphone, signifying another major niche for PHR expansion [43]. Select groups, such as the Children’s Partnership, have acknowledged that effective and accessible PHRs, when linked to a broader health information exchange system, could significantly increase the availability of accurate and timely information for young adults, especially those without guardians [44]. PHRs have the potential to facilitate better communication among providers, families, caseworkers, and others making care decisions on behalf of young people. Furthermore, PHRs also provide a mechanism for young people to become responsible for decisions regarding their own care and build self-sufficiency, particularly for those who lack access to regular medical care. Studies also indicate that PHRs also encourage parents to play more proactive roles in seeking out preventive medical care for their children; for example, parental PHR use is linked to improved immunization adherence [16].

In addition to mitigating the challenges associated with caring for pediatric populations, mPHRs may also be one of the only ways to quickly obtain patient information when medical facilities are struck by disaster and can no longer provide optimal care. As noted in one study, the value of PHRs “extends to contingency preparedness in all categories of events, from natural disasters to terrorist attacks, and infectious disease pandemics such as avian flu” [11]. As health care facilities move toward EHRs as their primary medical record systems, they rely more heavily on basic operational capabilities, such as electricity and functional emergency generators, to access the records stored on a facility’s server. When these assets fail, the quality of patient care, in turn, deteriorates. During Hurricane Sandy, for example, power outages and computer failures forced New York University Hospital to evacuate more than 200 patients (including critically ill infants) and transfer them to neighboring facilities [45]. Recordkeeping challenges often accompany such major transitions of care. Employees at Staten Island University Hospital, for instance, resorted to using paper records to track evacuated patients during Hurricanes Sandy and Irene, a strategy that proved inefficient and burdensome [46]. In such situations, mPHRs could assist health care providers in accessing patient information in a timely manner, thereby enabling them to make informed decisions and diagnoses that can reduce medical error and relay critical information for new patients. Furthermore, many cloud computing data centers often retain redundant power supplies, Internet connections, and hardware to ensure that consumers can still access their data even in the event of a power outage [47]. Providing more accurate and faster diagnoses is imperative when triaging patients and coordinating care during events that overwhelm health care facilities.

#### PHRs Have the Potential to Advance Telehealth

Telehealth, the delivery of health care via telecommunication technology, relies on methods such as real-time videoconferencing to facilitate teletriage and medical care in emergency scenarios. Telehealth strategies—videoconferencing, the use of smartphones and wireless networks, and email, for example—have already proven to be effective at sharing information between clinicians and medical facilities [48]. Select PHRs also enable information exchange between qualified clinicians, and can integrate transmission and virtual imaging capabilities. Thus, they offer a platform for “virtual visits,” allowing office-based health care providers and home-based health care workers or patients to coordinate patient management. Even basic patient information, such as blood pressure readings, temperature, glucose levels, and other medical notes, can be transmitted from home providers to physicians via a PHR. These functionalities enable physicians to obtain patient histories remotely and instruct home providers of necessary changes to the patient’s care regimen, which can be a key step toward building home telehealth capabilities. Integrating PHRs into a broader telehealth infrastructure could thus enhance emergency health care delivery by mitigating patient surges at health care facilities.

## Challenges

### Overview

Despite the potential benefits of integrating mPHRs into mainstream medical practice and disaster response efforts, some critical challenges remain. Because there are no documented cases of PHR usage in disaster response, it is difficult to identify the specific challenges associated with PHR implementation during PHEs, although several studies have explored the shifts in standards of care during disasters [48-51]. Furthermore, PHRs have yet to be widely adopted even in routine medical practice, suggesting that the barriers impeding increased use must be resolved first. These barriers, along with recurring, well-documented communication challenges during emergencies, are outlined below.

### The United States Lacks a Unified Infrastructure for Managing and Verifying the Integrity of Data Stored in PHRs

The ability to access accurate medical information is essential to the success of disaster response activities. However, the loss of critical infrastructure and personnel during PHEs impedes data collection. PHRs certainly present a viable solution to this problem, but require solid software platforms to function effectively. Most available PHRs and mPHRs currently operate on different, noninteroperable platforms, and thereby complicate efforts to gather pertinent medical information. Many PHR systems are integrated with a specific EHR system so, if EHR systems are not interoperable with each other, providers can be limited as to what content they can view. Furthermore, if PHR systems are stand-alone, and not integrated to a larger EHR system, users must manually enter information. Without the ability to exchange data with other systems, PHRs will devolve into “information islands” that remain divorced from other repositories of patient information, and provide limited value to health care professionals [52]. Such a lack of interoperability between EHR and PHR systems impedes information flow, which could be vital during emergencies. Standardization is also critical to the successful integration of PHRs into the nation’s HIT system.

Data integrity poses another critical challenge to clinicians relying on PHRs to access medical histories. Depending on the PHR service in question, clinicians may lack the ability to verify the accuracy of patient-entered data. Whereas PHRs provide useful patient information, consistent standards are needed to verify data integrity. For example, enabling consumer access, identifying who is entering the data, determining authenticity of data entered, and detailing how information flows from one PHR system to another or to an EHR system are necessary, minimum requirements to ensure overall interoperability of PHR systems.

Furthermore, in the event of emergencies, when electronic systems may fail or be vulnerable to external threats, EHR systems must be able to prevent unexpected loss of protected health information. If PHR systems become integrated into EHR systems, then PHR systems must also be able to manage potential threats. PHRs can provide necessary patient information when EHRs are unavailable, whether due to system hacking or power failures. PHRs are therefore the most viable “back up” option to provide individual patient information when original health records are destroyed or unavailable.

### Costs Associated With Maintaining PHRs Are Unclear and May Present a Significant Barrier to Medical Institutions

Although consumers have expressed interest in adopting PHRs to manage their health information, it remains unclear whether the cost of PHRs would dissuade potential users. Many PHRs are free, although select smartphone applications charge a nominal download fee. However, medical institutions seeking to establish PHR systems may face significantly higher costs. For example, one study considered the costs associated with developing an mPHR application, building the software infrastructure to run the application, and handling user activity. Their PHR model, which accounts for numerous costs during application development and authentication, including personnel costs, data centers, user support, and record matching services, generated costs of $450,000 [53]. Such expenses may be beyond the means of certain medical institutions aiming to provide their patients with cohesive, interoperable PHR platforms. In a different study, authors note that “many of the putative financial benefits of PHRs only occur when PHRs are tightly integrated with EHRs, so that seed funding of PHRs in practices that operate an EHR might advance PHR adoption to the ‘tipping point’” [52]. Similarly, another study points out that having “a common language with patients and readily available health information allows health care providers the potential to not only save lives, but to reduce the impact of the financial burden on our health care system” [54].

### Patient-Managed Health Records Present Consumers and Policymakers With Several Legal, Regulatory, and Privacy Challenges

As exemplified by the transition to EHRs, the health care industry in the United States is becoming increasingly reliant on technology. Consumers, too, are a major driving force behind the growing market for digital health care solutions. Despite these trends, however, legal and regulatory frameworks for governing mobile health care applications (as opposed to PHRs in non-mobile formats) remain conspicuously absent [6,35]. Given the continued expansion of mobile health, the current lack of regulation could impede health care delivery efforts in post-emergency settings. Previous PHR systems have encountered privacy and legal concerns, but the adaptation of PHRs into a mobile format, much like the adaptation of hospital records to EHRs, take on a new dimension of challenges regarding who can access and manipulate the data. PHEs, however, even those that complicate efforts to deliver timely, effective medical care, do not preclude the need for robust legal frameworks to protect patients. Resolving the challenges associated with maintaining protective legal standards would ensure patients’ privacy and safety even during PHEs. So far, legal and regulatory frameworks have generally failed to meet the challenges emerging from new developments and applications of consumer-operated, digital health care technologies. Three specific areas of concern include ownership and control of information in a PHR, third party use of consumer data for secondary purposes, and the application of existing laws to PHR systems.

PHRs vary in the way that consumers “own” their health information and, consequently, privacy and legal concerns vary according to the system’s design. Select PHRs give consumers access to their health information while others provide them exclusive access. Because of this difference in accessibility, insurance companies, health care providers, and others who administer PHRs must clarify the differences between legal control and ownership of the medical record. Moreover, relevant stakeholders must clarify how a consumer’s ownership and control over their own PHR and relevant personal health information differs from a provider or institution’s ownership and control over that same record. Such clarifications are needed to identify accountability for potential liabilities, rights to access, and obligations to act on data inputted into the PHR.

In addition to concerns surrounding control and ownership of PHR systems, privacy concerns also exist surrounding the use of third party vendors that may store or mine data for alternative uses. Several insurance companies and health care institutions may consider using, or already use third party systems for consumer data. However, it is unclear how these vendors use the consumer data in these systems. As PHR systems are designed and revised, consumers should be given the option to make their personal health information accessible to third party vendors for secondary uses.

### Roadblocks to the Integration of Personal Health Records and Electronic Health Records

PHRs allow iterative communication between patients and providers, and the integration of PHRs and EHRs would permit exporting data among information systems. An underlying challenge is that PHRs alone do not have universal standards and there is no standardized way to design and maintain PHRs [55]. Even if such challenges were resolved, and despite the potential benefits of integrating PHRs and EHRs, several factors would still inhibit their integration. First, it remains unclear how health system roles and responsibilities will change if systems are integrated. For example, concerns about liability risk and adverse effects for providers, such as increased workload and inadequate reimbursement, remain unresolved. Second, there is an absence of standards to inform the process by which systems should be integrated. Furthermore, it is unclear if there are limitations to the current HIT infrastructure that would present technical challenges to integration. Third, related to limitations of current infrastructure, concerns about privacy, security, the use of information by third parties, and lack of oversight impede integration [10,56].

## Recommendations

### Overview

Although the majority of PHR development in the United States takes place in the private sector, we maintain that the federal government is best suited to design, implement, and regulate PHR use during PHEs, given its involvement in emergency preparedness and response efforts at the national, state, regional, and local levels. The federal government also oversees health care delivery and innovation in HIT and is therefore well positioned to implement the appropriate standards for PHR standardization and interoperability. The following recommendations outline initial steps that federal agencies can take to integrate PHRs into the nation’s emergency response strategies and overall HIT infrastructure.

### Meaningful Use

Congress should identify meaningful use criteria to govern the use of mPHRs and allow eligible providers to earn incentive payments, modeled on the standards set for EHRs. Legislation that calls for the establishment of meaningful use criteria for mPHRs would encourage widespread adoption and use of mPHRs during emergency and nonemergency settings. Just as the American Recovery and Reinvestment Act of 2009 authorized incentivized payments for providers to encourage adoption and use of EHRs, such legislation could authorize incentive payments to providers that meet criteria for the use of mPHRs. Similarly, HHS’ Hospital Preparedness Program could help fund efforts to explore the role of public health records in augmenting surge responses during PHEs. Such criteria and incentives could help standardize how providers use mPHRs and direct relevant federal agencies to address challenges to their use.

Proposed legislation should specifically highlight the potential uses of mPHRs during emergencies. While many private companies develop and monitor mPHRs, establishing criteria for how providers and health care systems use and access these records can maximize the benefits of mPHRs to clinical outcomes.

### Integration Into Mainstream Health Care

Relevant federal entities, such as the Office of the National Coordinator for Health Information Technology (ONC), FDA, and HHS, should identify how to integrate PHRs and mobile portals into mainstream health care, especially for use during emergencies. Several challenges impede information flow among systems, such as difficulty integrating systems and monitoring how data from PHRs fit into EHRs. Aside from interoperability challenges, clear guidance may be needed to identify how and when health care providers should use data from mPHRs with routine clinical work. For example, it remains unclear if providers should receive payment for viewing data from mPHRs, if payment should be contingent on the situation in which an mPHR is used (ie, emergency versus nonemergency setting), and if providers will be held liable for actions determined according to the information in a PHR.

ONC and the Centers for Medicare & Medicaid Services have jointly developed meaningful use criteria for EHRs. These criteria would require EHRs to accept patient-generated data, which may include biometric data, blood pressure, information on chronic conditions and treatment, and other information in a patient’s medical history. For example, the Health Information Technology for Economic and Clinical Health Act of 2009 includes language describing meaningful use with respect to EHR technology, but lacks provisions for information exchange via mobile media [57]. Federal agencies involved in determining meaningful use criteria should clearly identify how patient-generated data should be used in broader HIT systems, to address issues of interoperability of systems and liability for providers.

Finally, the Hospital Preparedness Program, managed by the Assistant Secretary for Preparedness and Response, is charged with enhancing “hospital and health care system planning and response at the state, local, and territorial levels,” facilitating “the integration of public and private sector medical planning and assets to increase the preparedness, response, and surge capacity of hospitals and other health care facilities,” and improving “state, local, and territorial infrastructures that help hospitals and health care systems prepare for public health emergencies” [58]. Thus, exploring new modes of emergency communication and implementing crisis standards for information exchange certainly fall under the purview of HHS. HHS should therefore encourage health care providers, both private and public, to expand disaster response planning to integrate new communications technologies, including PHRs.

### Governance and Legislation

A federal agency, such as the ONC, should assume a regulatory role and establish a legal framework for mPHRs and related efforts. Many federal efforts have recognized the growth of the mobile health sector and identified the need for strategic leadership and guidance, yet critical gaps in existing legislation and governance remain. The absence of a dedicated regulatory body and legal framework for mobile health further exacerbates the challenges associated with expanding mPHR use. While the FDA has expanded guidance to include mobile apps that function as medical devices, guidance for managing patient information in post-emergency settings remains virtually nonexistent. Having these in place ensures that patients, clinicians, mobile application developers, and insurers are accountable for the management of mPHRs.

### Protection of Privacy

The application of existing legal and privacy provisions should be continuously addressed as mPHRs develop. Because PHRs and mPHRs are emerging health information technologies, the legal and privacy concerns regarding their use may change as technologies and their roles in HIT more generally evolve. Select PHRs, offered by health care providers and health plans, are covered by the Health Insurance Portability and Accountability Act of 1996 (HIPAA) Privacy Rule. In the event that HIPAA applies to a PHR or mPHR, the information within these records is protected by the law. However, those systems that are not offered by HIPAA covered entities must adhere to the privacy policies and their respective vendors. A system that is not covered by HIPAA may be covered by other applicable laws; however, all PHR and mPHR system providers should identify how health information is protected and communicate such policies to consumers.

### Marketplace Support

Congress should identify new ways to support HIT development. In 2012, Representative Mike Honda introduced the Healthcare Innovation and Marketplace Technologies Act (HIMTA), aimed at fostering further innovation and entrepreneurship in the HIT sector. Congress should support the legislation, and similar bills, that calls on the FDA to establish a marketplace for new technologies and advance training for health care workers to handle their implementation and use [26,59]. HIMTA specifically contains provisions for an FDA-run Office of Wireless Health that will create and maintain the necessary framework to regulate mobile health technologies, such as mPHRs. The office would also offer legal support to mobile developers, provide loans to physicians who adopt new technologies, and create educational materials to elucidate privacy guidelines [60]. Despite the potential benefits of enacting HIMTA, the FDA maintains that its forthcoming mobile medical application policies would not apply to mobile applications that perform the functions of an HER or PHR system [61]. Congress should therefore encourage the FDA and other relevant federal agencies to identify their respective roles in supporting the development of PHRs and similar forms of HIT. Congress should also develop crisis standards of operation for technologies that facilitate health information exchange during PHEs.

## Abbreviations

CDC: Centers for Disease Control and Prevention
ED: emergency department
EHR: electronic health record
EMR: electronic medical record
FDA: US Food and Drug Administration
HHS: US Department of Health and Human Services
HIMTA: Health Innovation and Marketplace Technologies Act
HIPAA: Health Insurance Portability and Accountability Act
HIT: health information technology
mPHR: mobile personal health record
ONC: Office of the National Coordinator for Health Information Technology
PHE: public health emergency
PHR: personal health record
